# Association between Dietary Diversity and All-Cause Mortality: A Multivariable Model in a Mediterranean Population with 18 Years of Follow-Up

**DOI:** 10.3390/nu14081583

**Published:** 2022-04-11

**Authors:** Laura Torres-Collado, Manuela García-de la Hera, Naomi Cano-Ibañez, Aurora Bueno-Cavanillas, Jesús Vioque

**Affiliations:** 1 Instituto de Investigación Sanitaria y Biomédica de Alicante, ISABIAL-UMH, 03010 Alicante, Spain; l.torres@umh.es (L.T.-C.); vioque@umh.es (J.V.); 2 Unidad de Epidemiología de la Nutrición, Departamento de Salud Pública, Historia de la Ciencia y Ginecología, Universidad Miguel Hernández (UMH), 03550 Alicante, Spain; 3 CIBER Epidemiología y Salud Pública (CIBERESP), Instituto de Salud Carlos III, 28034 Madrid, Spain; ncaiba@ugr.es (N.C.-I.); abueno@go.ugr.es (A.B.-C.); 4 Department of Preventive Medicine and Public Health, University of Granada, 18016 Granada, Spain; 5 Instituto de Investigación Biosanitaria (ibs.GRANADA), 18014 Granada, Spain

**Keywords:** dietary diversity score, mortality, cardiovascular disease, cancer

## Abstract

We evaluated the relationship between the dietary diversity score (DDS) and all-cause, CVD and cancer mortality in an adult Mediterranean population. We analyzed the data of 1540 participants from the Valencia Nutrition Survey. The DDS was estimated using a validated food frequency questionnaire and was categorized into quartiles (Q), where the first quartile indicates the lowest dietary diversity. Deaths were ascertained during an 18-year follow-up period. Cox regression models were used to estimate hazard ratios (HR) and 95% confidence intervals (CI). There were 403 deaths during the follow-up period (40% due to CVD). An inverse association was observed between the DDS and all-cause and CVD mortality. Compared with participants in the lowest DDS quartile (Q1), participants in the highest DDS quartile (Q4) showed 32% and 45% less risk of death for all-cause and CVD mortality, in sex- and age-adjusted models, respectively. Regarding the food groups in the DDS, an inverse association was identified between total vegetable consumption diversity and all-cause and CVD mortality in the highest quartiles, (Q3 vs. Q1, HR: 0.70; 95% CI: 0.50, 0.99) and (Q4 vs. Q1, HR: 0.52; 95% CI: 0.30, 0.91), respectively. This study suggests that a higher diversity in food intake, particularly in vegetables, may be associated with a lower risk of all-cause and CVD mortality. This association should be further investigated in other wider populations.

## 1. Introduction

Diet is one of the major determinants of health. During recent years, the disease burden due to poor diet quality has increased worldwide, causing more than 11 million deaths [1]. Previous evidence has shown that dietary scores can measure the effects of overall diet quality in relation to the prevention of chronic diseases or premature cardiovascular mortality or morbidity [2]. One widely used approach to examine total diet quality is to evaluate dietary diversity. The dietary diversity score (DDS) is a simple and global indicator, recommended in the dietary guidelines of many countries [3,4], which evaluates the variety of several food groups (i.e., fruits, vegetables, proteins, dairy, and grains), [5,6] according to the nutritional recommendations. This score has been considered as an index of diet quality, mainly due to its correlation with nutrient adequacy [7,8,9].

A higher DDS has been associated with a lower incidence of diabetes [10], metabolic syndrome [11], cardiovascular disease (CVD) [12,13,14], and all-cause mortality [15,16,17]. Regarding the relationship between all-cause mortality and the DDS, the majority of the studies that included older populations showed an inverse association [15,16,17]. In adult populations, the prospective study of the first National Health and Nutrition Examination Survey (NHANES) showed that a low DDS was associated with an increased risk of all-cause mortality [18,19]. These findings are in line with the results found by Kobayashi et al. [20] in a recent study that enrolled 79,904 participants (37,240 men and 42,664 women), aged 45 and above, who were followed up for a median follow-up period of 14.9 years. However, while this study showed an inverse association between a high total DDS and all-cause mortality in women (HR: 0.81; 95% CI: 0.71–0.92), these results were not observed in men (HR: 0.96; 95% CI: 0.87–1.10) [20].

To date, little is known regarding the association between the DDS and CVD and cancer mortality. To the best of our knowledge, only two studies have evaluated these associations in adult populations [19,20]. Kant et al., showed a higher risk of CVD mortality in lower DDS categories, although this association was attenuated in the more fully adjusted models. Kobayashi et al., showed that women in the higher DDS category showed lower risk of CVD mortality (HR: 0.66; 95% CI: 0.51–0.86). These two studies also found some evidence of a protective effect for cancer mortality in the highest DDS categories, although the associations were not statistically significant [19,20].

Hence, a study on the association between the DDS and mortality is justified, especially in Mediterranean populations with high life-expectancy and healthy diets, for which no previous evidence exists. Thus, the present study aimed to evaluate the association between the DDS and all-cause, CVD and cancer mortality in an adult Mediterranean population aged 20 and above in Spain.

## 2. Materials and Methods

### 2.1. Study Population

We analysed data from 1540 participants (718 men and 822 women, aged 20–97 years old) enrolled in the Valencia Nutrition Survey (VNS) conducted in 1994. Survey methods have been described in detail elsewhere [21]. Briefly, the VNS is a survey which enrolled 1811 participants (74.4% participation rate) and was designed to investigate the health and nutrition status of the adult population in the Valencia Region aged 15 years and older. Participants with no information regarding diet and those younger than 20 years were excluded from the present analysis. In addition, we did not include participants with an intake lower than 800 kcal/day or higher than 4000 kcal/day, as they were considered as implausible intakes [22]. Thus, the final sample for this analysis was conducted with 1540 participants aged 20 years and above with complete information.

We obtained written informed consent from all participants. The study followed the principles of the declaration of Helsinki and was approved by the Ethical Committees of the Miguel Hernandez University (Spain).

### 2.2. Dietary Assessment

In order to assess the dietary information, we used a semiquantitative FFQ of 93 food items, which had a similar structure to the Willett questionnaire and was previously adapted and validated in adult populations in Spain [23,24,25]. The FFQ included ten categories for the main food groups: vegetables, fruits, meat and fish, eggs, dairy, oils and fats, breads and cereals, sweets and pastry, processed foods, and beverages. The FFQ showed satisfactory reproducibility and validity when we compared food intake and nutrient estimates in the adult population with those from four one-week dietary records [24,25,26,27]. The average correlation coefficients for one-year reproducibility and validity of nutrient intakes were 0.40 and 0.47, respectively, which denote a satisfactory validity of the FFQ for dietary assessment, in line with the range 0.30–0.70 observed in literature [22]. We have also validated our FFQ in other adult populations showing acceptable biochemical validity [26,27]. We are aware that there is no perfect method to assess diet and some misclassification is present when using FFQ, although it is assumed it should not be differential, biasing the associations toward the null, if any.

We asked participants to report the average frequency of consumption for the specified standard portion size or serving for each food item during the previous year. The FFQ had nine possible frequency options, from “never or less than once a month” to “six or more times a day”. To estimate nutrient values and total energy intake, we used the published food composition tables of the US Department of Agriculture (USDA) and other published sources for specific Spanish food and portion sizes [28,29]. In order to obtain the average daily nutrient intakes, we multiplied the frequency of consumption of each food item by the nutrient composition of the serving size specified and added the results for each food.

### 2.3. Dietary Diversity Score Construction

We calculated the DDS using the original method developed by Kant et al. [5], which has been used in other recent studies [13,30]. The DDS was based on five groups: fruits, vegetables, dairy products, cereals, and proteins. These five groups were created according to the recommendations of the Spanish nutritional pyramid guidelines [31]. In Table 1, we detailed food groups included in the DDS and the recommended servings per/day. We did not include non-recommended food groups in the analysis, as they are considered unhealthy [32,33]. These unhealthy foods are characterized by high salt and saturated fat content (butter, margarine, red meat, processed meats, sauces, pre-cooked dishes, condiments, and snacks) or sugar (pastries, pies, biscuits, chocolate, fruit in syrup, and fruit juices).

The Spanish nutritional pyramid guidelines define optimal dietary diversity as the consumption of at least half of the recommended servings per day for each food group [31]. Thus, if a subject consumes at least half of the recommended serving, the score will be 2 points, while consumption of less than half of the recommended serving will score 0. For example, if the Spanish recommendations for vegetables are 2 servings per day, each individual who consumes at least 1 serving per day will score 2 points while, an individual who consumes less than 1 serving per day will score 0.

The score for each group can range from 0 to a maximum of 2 points. The procedure is the same for each food group considered. In short, to calculate each group, we added all food components belonging to this food group and then we multiplied by two. Finally, we divided this result by the total number of items that were included in this group. Thus, the total DDS, which can range between 0 (minimum) and 10 points (maximum), was calculated by combining the scores of these five groups.

We adjusted the DDS for the total energy intake using the residual method as recommended by Willett et al. [22]. Finally, the DDS was categorized into quartiles (Q), and similarly, each food group was classified into four categories (C). The cut-off points for the DDS were: Q1: 4.5; Q2: 5.4; Q3: 6.1; Q4: 8.9. Finally, the cut-off points for each food group were: C1 = 0 points, C = >0 to <1.0 point, C3 = 1.0 point and C4 = ≥1.5 points; for cereal group were: C1 = <1.0 point, C2 = 1.0 point, C3= 1.5 points and C4 = ≥2.0 points; for dairy products were: C1 = 0 points, C2 = >0 to 1.0 point, C3 = <2.0 point and C4 = ≥2.0 points; and for proteins were: C1= ≤0.5 points, C2 = ≤0.8 points, C3 = ≥1.2 points and C4 = ≥1.6 points.

### 2.4. Mortality Assessment

During the 18-year follow-up period, date and specific cause of death were verified through the National Death Index from the Spanish Statistical Office and the Mortality Registry in the Valencia Region.

We coded all causes of death using version 10 of the International Classification of Diseases (ICD-10). We classified deaths into three broad categories: cardiovascular disease (ICD-10: I00-I99), cancer (ICD-10: C00-D49), and all-cause mortality that included deaths from any cause.

### 2.5. Other Variables

At baseline, trained fieldworkers asked all participants about their sociodemographic characteristics and lifestyles using structured questionnaires. We considered the following variables in the analyses: sex (men, women), age (in years), educational level (<primary school; >primary school), body mass index (BMI) measured as weight in kilograms divided by the square of measured height in meters (<25 kg/m^2^, 25–30 kg/m^2^, >30 kg/m^2^), smoking habit (never, ex-smoker, current), total hours of TV watching per day, and total sleeping time in hours per day. We also collected the presence of chronic disease at baseline, such as diabetes (no/yes) and high blood pressure (no/yes). In adult populations, self-reported diseases and those documented in medical records have shown a high level of concordance [34,35].

### 2.6. Statistical Analysis

We carried out a descriptive analysis of sociodemographic characteristics using n, percentages and χ^2^ tests to describe and compare categorical variables. For continuous variables, means, standard deviations, and ANOVA with Bonferroni post-hoc tests were used.

We calculated person-years for each subject from the date of the interview at baseline to the date of death or completion of the 18-year follow-up, whichever came first. Hazard ratios (HRs) and 95% confidence intervals (95% CI) were obtained using Cox’s proportional hazard models for each category of the DDS from all-cause, CVD, and cancer mortality.

For the presentation of the results, we ran two models in which we further adjusted for several factors considered as potential confounders by the literature. We also adjusted models with variables showing *p*-values < 0.20 in bivariate analysis. Model 1 was adjusted for age and sex, and Model 2 was adjusted for the variables in model 1 plus education level (<primary school; ≥primary school), BMI (<25 kg/m^2^, 25–30 kg/m^2^, >30 kg/m^2^), tobacco (never, ex-smoker, current), alcohol intake (g/day), TV watching (hours per day), total sleeping time (hours per day), presence of diabetes (no/yes), and high blood pressure (no/yes).

Finally, the likelihood ratio test (LRT) was used to evaluate the overall significance of the DDS considered as a categorical variable. We also used LRT with one degree of freedom to evaluate the presence of linear dose-response for categorical variables as a continuous term (trend test, *p*-trend), with each model including all potential confounders. The level of statistical significance was set at 0.05 and all tests were two-tailed.

All analyses were performed using STATA^®^, Version 16, StataCorp LP, College Station, TX, USA.

## 3. Results

The participants’ main baseline characteristics according to the four categories of the DDS are presented in Table 2. Participants with a higher DDS were more likely to be women, older, never smoked and had lower alcohol consumption. Out of 1540 participants, 385 (25.0%) were in the highest DDS quartile.

**Figure 1:**
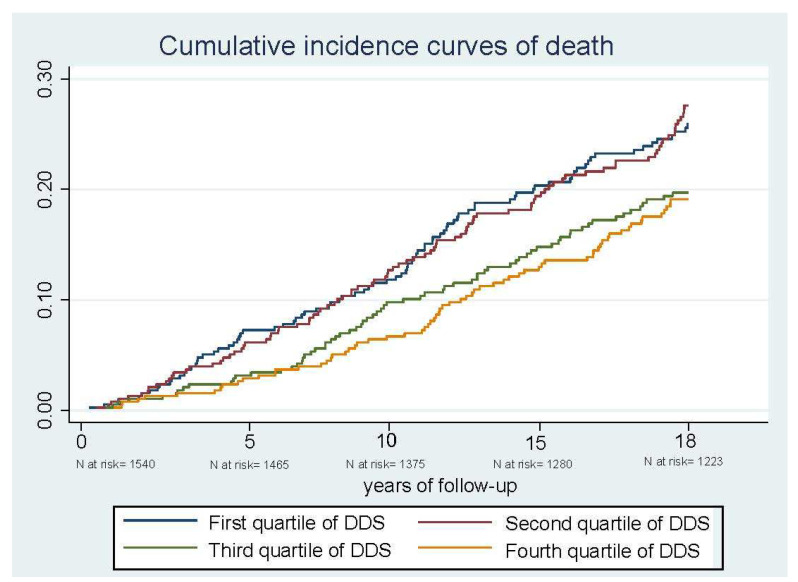
Cumulative incidence curves of death after 18 years of follow-up, according to Dietary Diversity Score (DDS) for all-cause mortality in participants from the Valencia Nutritional Survey in Spain.

Table 3 shows the number of deaths and hazard ratios according to the categories of the DDS. During the 18 years of follow-up (9169.5 person-years), we documented 317 deaths, 115 of which were due to CVD (36.3%) and 82 to cancer (25.9%). At 18 years of follow-up, we observed that participants with a high DDS had lower cumulative incidence curves for all-cause mortality than those with the lowest DDS (Figure 1). The DDS showed an inverse association with all-cause and CVD mortality (Table 3). Compared with participants in the lowest DDS quartile, participants in the highest DDS quartile showed 32% less risk of death for all-cause mortality (HR: 0.68; 95% CI: 049–0.94) in sex- and age-adjusted models. In addition, these participants had 21% less risk of death in the multivariable model (HR: 0.79; 95% CI: 0.57–1.10), although this association was not statistically significant.

A lower CVD mortality was also observed among participants in the highest DDS quartile. The age- and sex- adjusted HR was (HR: 0.55; 95% CI: 0.32–0.93), although this association lost significance in the fully adjusted model (HR: 0.61; 95% CI: 0.35–1.07). We did not observe significant associations for cancer mortality.

The association between each food group included in the DDS and all-cause, CVD and cancer mortality is shown in Table 4. Compared with participants in the lowest quartile, a lower all-cause mortality was observed with vegetable consumption among participants in the highest quartiles of this food group. Participants in the third and fourth quartiles of vegetable consumption showed a 29% and 30% less risk of all-cause mortality, (HR: 0.71; 95% CI: 0.51–0.98) and (HR: 0.70; 95% CI: 0.50–0.99), respectively. Similarly, we observed 45% and 48% less risk of CVD mortality, respectively, in participants in the second and fourth quartiles of vegetable consumption. Overall, no statistically significant associations were found between fruits, cereals, dairy products or proteins and all-cause, CVD and cancer mortality at 18 years of follow-up.

## 4. Discussion

This study showed an inverse association between DDS and all-cause and CVD mortality in the age- and sex-adjusted models, although these associations were attenuated and lost statistical significance in more fully adjusted models. When we explored this association by food group components of the DDS, it was only in the highest quartile of vegetable consumption that we found a lower risk for all-cause and CVD mortality. Non-significant associations were observed for cancer mortality.

Some studies reported inverse associations between a high DDS and all-cause mortality, but most of these studies have been carried out in older Japanese and Chinese populations [15,16,17,36]. Lv et al., evaluated this association in 28,790 Chinese participants aged 80 years and above and showed that, compared to participants with a lower DDS, those with the highest DDS had 44% less mortality risk [17]. Along the same line, Otsuka et al., observed that participants in the highest DDS tertiles had 31% less risk of all-cause mortality compared to the participants in the lowest tertiles. Few studies have evaluated the association in adult populations. To the best of our knowledge, only one study has evaluated the association between the DDS and all-cause, CVD or cancer mortality in adult populations. The Japan Public Health Center (JPHC)-based prospective study showed an inverse association between total DDS and all-cause and CVD mortality in women in the highest quintile, but these associations were not observed in men [20].

Our results are consistent with the results of the Japanese study carried out in adult populations [20]. We observed 32% lower all-cause mortality and lower 45% CVD mortality among participants in the highest quartile of the DDS compared to those in the lowest quartile in the adjusted sex-age model. However, these effects were not statistically significant in multivariable models. Another study supported our findings. The results of the NHANES I Epidemiologic follow-up study showed strong significant associations in sex and age models which were attenuated in the multivariable models. This attenuation of effect might be related to several factors, including the lack of statistical power due to the low number of deaths during the follow-up period. Moreover, the DDS, like other scores based on individual dietary assessment methods, are subject to measurement error due to within subject variability regarding food intake [19,37]. Despite diet being a habit that remains quite stable over time [38,39], it was assessed at baseline in our study, and some individuals might have changed their diets during follow-up. Thus, it could result in some misclassification of participants’ responses when categorizing usual dietary habits and tends to attenuate the associations between DDS and all-cause and CVD mortality. In addition, the fact that the DDS is an index based on the five major food groups and measures the minimum recommended amount intake rather than quantitative estimates of food intake can also influence this attenuation.

Few studies have assessed dietary diversity according to intake of the main food groups that have been consumed per day, and all-cause, CVD and cancer mortality [15,16,17,20]. In our study, we only observed significant inverse associations between all-cause and CVD mortality with the highest quartile of vegetable consumption diversity, which does not necessarily mean a high consumption of vegetables in quantitative terms as directly calculated in other studies exploring the association with vegetable intake. In relation to all-cause mortality, we observed that those in the third and fourth quartiles had 29% and 30% less risk of all-cause mortality. Some studies explored the association between vegetable consumption diversity and all-cause mortality but found no association [15,20]. Our results are in line with a Chinese study that showed 12% and 6% lower risk of all-cause mortality in older and octogenarian populations who consume the recommended level of vegetable consumption diversity [16,17].

Regarding the association with CVD mortality, our results showed that compared to the lowest quartile, those in the second and fourth quartiles had 45% and 48% less risk of CVD mortality. As far as we know, only one study has evaluated the association between the diversity of consumption of specific food groups and CVD mortality. Kobayashi et al. [20] showed a positive non-significant association in participants with the highest vegetable consumption diversity; the lack of significance in this study could be due, in part, to misclassification in reporting the consumption of different types of cooked or preserved vegetables together with other foods. We should also consider that food intakes may be influenced by the way they are cooked in Japan and Spain and by the type of fat used for dressing or cooking vegetables. We could not address this issue in more depth in our study.

Our findings provide some evidence that the highest DDS and particularly the highest intake of the vegetable group, a key component of DDS, may be associated with less all-cause and CVD mortality in adult Mediterranean populations. If confirmed by other studies, these results could help to develop new recommendations to increase dietary diversity, mainly by promoting a higher diversity of vegetable intake.

Finally, regarding the effect of the DDS and some food components on cancer mortality, we observed non-significant positive associations with DDS, dairy products, and proteins, probably because our sample size was too limited to evaluate these associations properly.

Our study has several limitations that should be discussed. First, we evaluated usual dietary intake at baseline that might have changed during follow-up, despite previous studies reporting that diet is a habit that remains stable over time and diet assessed at baseline might be valid in order to evaluate long-term dietary exposure in nutritional epidemiological studies [38,39]. Thus, we assumed that a change in diet after baseline, if any, should be non-differential, reinforcing our study results for the DDS and vegetables. Second, we used a FFQ validated in a Spanish population, although misreporting of food intakes could have caused some non-differential misclassification bias that would tend to bias the HR toward the null, if any. Third, we calculated the DDS excluding non-recommended food products with a high content of sugar or saturated fats, and red or processed meats using the methodology described by Kant et al. [5]. Nevertheless, we considered that the DDS would not increase with the consumption of any of these non-recommended food products. Fourth, participants in our nutrition survey were volunteers and some response bias is possible, although this is improbable because usual dietary intake is unlikely to have influenced the participation rate in our study. Fifth, we must recognize that the associations we found for the DDS and mortality in our study were not based on statistically significant LRTs, although we found evidence of significant association for the highest categories of dietary diversity, and particularly, for the vegetable group with all-cause and CV mortality, for which we also found significant dose-response. Lastly, the sample size was not estimated a priori for this survival study, since the available data were from the Nutrition Survey of Valencia carried out in the nineties on a representative sample of adult population of Valencia Region. This survey had the aim to describe distributions and detect differences in the main lifestyles and dietary variables.

Our study also has some strengths. It involved a well-defined population which included subjects aged 20 years and above from a Mediterranean area. In addition, trained fieldworkers collected high quality information about dietary intake and sociodemographic characteristics at baseline, using standardized and validated questionnaires. Thus, any misclassification occurring in relation to the DDS categories should be non-differential in essence.

## 5. Conclusions

In summary, the present study suggests that a high vegetable consumption diversity is associated with a lower all-cause and CVD mortality after 18 years of follow-up. We also observed evidence that a higher DDS might reduce all-cause and CVD mortality, although our study was unpowered to detect these associations as significant formally considering the LRT. Thus, further prospective studies are recommended to confirm our results and increase evidence about the relationship between high-food diversity consumption and mortality in adult populations.

## Figures and Tables

**Table 1:** Food groups and the recommended servings per day/week used in the dietary diversity score (DDS) according to the Spanish guidelines.

Food Groups	Food Subgroups	Recommended Servings
Vegetables	(1) Green vegetables: spinach, cauliflower, lettuce, green beans, eggplant, peppers, asparagus; (2) Tomatoes; (3) Yellow vegetables: carrots, onion; (4) Mushrooms.	2 servings/day
Fruits	(1) Citrus fruits: orange, apple; (2) Tropical Fruits: banana, fig, grapes; (3) Seasonal fruits: strawberries, cherry, peach; (4) Other seasons fruits: watermelon, melon.	3 servings/day
Dairy products	(1) Milk: low fat and high fat; (2) Yogurt; (3) Cheese: curd, cream, cured.	2 servings/day
Cereals	(1) Potatoes; (2) Pasta; (3) Rice; (4) Bread.	4 servings/day
Proteins	(1) Legumes; (2) White meats: poultry and rabbit; (3) Fish: oily fish, white fish and other shellfish/seafood; (4) Eggs; (5) Nuts.	3 servings/week

**Table 2:** Sociodemographic and lifestyle characteristics according to dietary diversity score among participants of the Valencia Nutrition Study in Spain (*n* = 1540).

		Dietary Diversity Score				
	Total	Q1 (≥1.8, <4.5)	Q2 (≥4.5, <5.4)	Q3 (≥5.4, <6.1)	Q4 (≥6.1, <8.9)	*p*-Value ^1^
DDS, n (%)	1540	385 (25.0)	385 (25.0)	385 (25.0)	385 (25.0)	
Sex = women, n (%)	822 (53.4)	163 (42.3)	190 (49.3)	215 (55.8)^3^	254 (66.0) ^3^	<0.001
Age, mean (SD)	46.1 (18.1)	44.3 (19.2)	46.6 (19.1)	46.3 (17.2)	47.4 (16.6)	0.11
Education Level, n (%)						
<Primary school	701 (45.5)	175 (45.4)	176 (45.7)	177 (46.0)	173 (45.0)	0.99
≥Primary school	839 (54.5)	210 (54.6)	209 (54.3)	208 (54.0)	212(55.0)	
Body Mass Index (kg/m^2^), n (%)						
<25 kg/m^2^	635 (41.5)	168 (43.9)	152 (39.9)	165 (43.0)	150 (39.9)	0.83
25–30 kg/m^2^	615 (40.1)	145 (38.0)	158 (41.5)	150 (39.0)	162 (42.1)	
>30 kg/m^2^	282 (18.4)	69 (18.1)	71 (18.6)	69 (18.0)	73 (19.0)	
Smoking Status, n (%)						
Never	763 (49.5)	159 (41.3)	172 (44.7)	208 (54.0) ^3^	224 (58.2) ^3^	<0.001
Former	260 (16.9)	66 (17.1)	62 (16.1)	62 (16.1)	70 (18.2)	
Current	517 (33.6)	160 (41.6)	151 (39.2)	115 (29.9) ^3^	91 (23.6) ^3^	
Diabetes ^2^ (yes), n (%)	120 (7.8)	28 (7.3)	34 (8.8)	37 (9.6)	21 (5.5)	0.14
Hypertension ^2^ (yes), (n %)	280 (18.2)	63 (16.4)	73 (19.0)	65 (16.9)	79 (20.5)	0.41
Alcohol consumption, mean (SD)	8.5 (16.1)	12.5 (21.6) ^3^	8.9 (16.6) ^3^	7.0 (13.4) ^3^	5.8 (9.9) ^3^	<0.001
TV-watching, hours/day, mean (SD)	2.5 (1.8)	2.6 (1.9)	2.5 (1.6)	2.5 (1.8)	2.3 (1.6)	0.07
Sleeping time, hours/day, mean (SD)	7.5 (1.4)	7.4 (1.5)	7.4 (1.4)	7.4 (1.3)	7.4 (1.3)	0.88

Abbreviations: SD, Standard deviation; BMI, Body Mass Index; TV, television. ^1^ *p*-value from chi-square test (categorical variables) and ANOVA (continuous variables). ^2^ Self-reported diabetes (no/yes), high cholesterol (no/yes), and hypertension (no/yes). ^3^ These categories were statistically significant from the others in post-hoc Bonferroni test.

**Table 3:** Associations between dietary diversity score and all-cause, cardiovascular disease and cancer mortality among participants of the Valencia Nutrition Study in Spain (*n* = 1540).

	Dietary Diversity Score					
	Q1 (≥1.8, <4.5)	Q2 (≥4.5, <5.4)	Q3 (≥5.4, <6.1)	Q4 (≥6.1, <8.9)	*p*-Value ^2^	*p*-Trend ^3^
	Follow-up at 18 years					
All-cause (*n*, %)	385 (25.0)	385 (25.0)	385 (25.0)	385 (25.0)		
Deaths, *n*	88	93	69	67		
Person-years	6120.2	6145.6	6349.4	6425.8		
HR (95% CI)						
Age and sex adjusted	1.00	0.85 (0.63–1.13)	0.71 (0.51–0.97)	0.68 (0.49–0.94)	0.07	0.01
Multivariable ^1^	1.00	0.87 (0.64–1.17)	0.72 (0.51–1.00)	0.79 (0.57–1.10)	0.23	0.09
CVD (*n*, %)	330 (24.7)	324 (24.2)	341 (25.5)	343 (25.6)		
Deaths, *n*	33	32	25	25		
Person-years	5657.1	5560.7	5941.0	5983.6		
HR (95% CI)						
Age and sex adjusted	1.00	0.74 (0.45–1.21)	0.64 (0.38–1.07)	0.55 (0.32–0.93)	0.14	0.02
Multivariable ^1^	1.00	0.81 (0.48–1.35)	0.71 (0.41–1.22)	0.61 (0.35–1.07)	0.37	0.08
Cancer (*n*, %)	316 (24.1)	320 (24.5)	334 (25.6)	335.25.7		
Deaths, *n*	19	28	18	17		
Person-years	5490.1	5513.5	5880.5	5889.8		
HR (95% CI)						
Age and sex adjusted	1.00	1.26 (0.70–2.26)	0.86 (0.45–1.66)	0.74 (0.38–1.45)	0.35	0.21
Multivariable ^1^	1.00	1.34 (0.73–2.45)	0.99 (0.50–1.95)	1.05 (0.52–2.13)	0.40	0.90

Abbreviations: CI: confidence interval; CVD: cardiovascular disease. ^1^ Cox regression model adjusted for age (continuos), sex, educational level (<Primary, ≥Primary), BMI (<25, 25.0–29.9, ≥30), sleeping time (hours/day), smoking habit (current, past, and never), self-reported diabetes (no/yes), high cholesterol (no/yes), hypertension (no/yes), and TV-watching (hours/day).^2^ *p*-value from likelihood ratio test. ^3^ The level of statistical significance was set at 0.05, a *p*-trend < 0.05 was suggestive of a linear monotonic trend.

**Table 4:** Associations between food groups of the dietary diversity score and all-cause, cardiovascular disease and cancer mortality among participants of the Valencia Nutrition Study in Spain (*n* = 1540).

	Food Components of the DDS					
					*p*-Value ^2^	*p*-Trend
Fruit group	C1 (0.0 *p*)	C2 (0.0; <1.0 *p*)	C3 (1.0 *p*)	C4 (≥1.5 *p*)		
All-cause mortality, (*n*, %)	250 (16.2)	576 (37.4)	431 (28.0)	283 (18.4)		
Deaths; person-years	37; 4132.9	123; 9282.9	98; 7036.5	59; 4588.6		
Multivariable ^1^	1.00	0.99 (0.68–1.44)	0.97 (0.66–1.42)	1.13 (0.73–1.72)	0.84	0.60
CVD (*n*, %)	223 (16.7)	505 (37.7)	368 (27.5)	242 (18.1)		
Deaths; person-years	10; 3909.8	52; 8645.5	35; 6371.6	18; 4215.4		
Multivariable ^1^	1.00	0.94 (046–1.90)	0.98 (0.48–2.02)	0.85 (0.37–1.90)	0.96	0.75
Cancer (*n*, %)	226 (17.3)	482 (36.9)	358 (27.4)	239 (18.3)		
Deaths; person-years	13; 3943.4	29; 8387.9	25; 6266.9	15; 4175.7		
Multivariable ^1^	1.00	0.76 (0.38–1.51)	0.83 (0.42–1.68)	0.97 (0.45–2.09)	0.83	0.88
Vegetable group	C1 (0.0 *p*)	C2 (0.0; <1.0 *p*)	C3 (1.0 *p*)	C4 (≥1.5 *p*)		
All-cause mortality, (*n*, %)	285 (18.5)	372 (24.2)	479 (31.1)	404 (26.2)		
Deaths; person-years	68; 4480.5	82; 6059.3	91; 7867.5	76; 6633.7		
Multivariable ^1^	1.00	0.79 (0.57–1.11)	0.71 (0.51–0.98)	0.70 (0.50–0.99)	0.16	0.04
CVD (*n*, %)	247 (18.5)	317 (23.7)	421 (31.5)	353 (26.4)		
Deaths; person-years	30; 4173.9	27; 5519.8	33; 7279.2	25; 6169.4		
Multivariable ^1^	1.00	0.55 (0.31–0.96)	0.63 (0.37–1.07)	0.52 (0.30–0.91)	0.09	0.05
Cancer (*n*, %)	233 (17.8)	316 (24.2)	405 (31.0)	351 (26.9)		
Deaths; person-years	16; 4039.3	26; 5458.9	17; 7153.8	23; 6121.9		
Multivariable ^1^	1.00	1.23 (0.64–2.37)	0.59 (0.29–1.18)	0.79 (0.40–1.50)	0.12	0.14
Cereal group	C1 (<1.0 *p*)	C2 (1.0 *p*)	C3 (1.5 *p*)	C4 (≥2.0 *p*)		
All-cause mortality, (*n*, %)	418 (27.1)	546 (35.4)	403 (26.2)	173 (11.2)		
Deaths; person-years	86; 6745.9	124; 8788.6	79; 6645.3	28; 25,040.9		
Multivariable ^1^	1.00	0.98 (0.74–1.31)	1.03 (0.75–1.42)	1.10 (0.71–1.71)	0.95	0.66
CVD (*n*, %)	360 (26.9)	472 (35.3)	350 (26.2)	156 (11.7)		
Deaths; person-years	28; 6236.4	50; 8095.5	37; 6100.4	11; 2709.9		
Multivariable ^1^	1.00	1.09 (0.67–1.76)	0.84 (0.47–1.46)	1.56 (0.76–3.19)	0.41	0.76
Cancer (*n*, %)	359 (27.5)	450 (34.5)	347 (26.6)	149 (11.4)		
Deaths; person-years	27; 6217.5	28; 7839.4	23; 6065.7	4; 2651.4		
Multivariable ^1^	1.00	0.83 (0.47–1.43)	1.11 (0.62–1.99)	0.68 (0.23–1.99)	0.66	0.90
Dairy group	C1 (0.0 *p*)	C2 (≤1.0 *p*)	C3 (<2.0 *p*)	C4 (>2.0 *p*)		
All-cause mortality, (*n*, %)	143 (9.3)	696 (45.2)	571 (37.1)	130 (8.4)		
Deaths; person-years	41; 2228.8	171; 11,034.0	86; 9570.5	19; 2207.6		
Multivariable ^1^	1.00	1.08 (0.76–1.55)	0.87 (0.59–1.28)	1.07 (0.61–1.90)	0.40	0.41
CVD (*n*, %)	118 (8.8)	581 (43.4)	522 (39.0)	117 (8.7)		
Deaths; person-years	16; 2012.6	56; 9944.8	37; 9116.3	6; 2068.6		
Multivariable ^1^	1.00	0.94 (0.52–1.72)	0.75 (0.40–1.39)	1.04 (0.39–2.80)	0.67	0.44
Cancer (*n*, %)	112 (8.6)	571 (43.7)	507 (38.8)	115 (8.8)		
Deaths; person-years	10; 1923.3	46; 9888.1	22; 8931.3	4; 2031.4		
Multivariable ^1^	1.00	1.02 (0.51–2.07)	0.80 (0.37–1.71)	1.04 (0.31–3.45)	0.81	0.55
Protein group	C1 (≤0.5 *p*)	C2 (0.8 *p*)	C3 (≥1.2 *p*)	C4 (≥1.6 *p*)		
All-cause mortality, (*n*, %)	170 (11.0)	378 (24.5)	608 (39.5)	384 (24.9)		
Deaths; person-years	64; 2507.3	88; 6067.8	122; 9910.0	43; 6555.7		
Multivariable ^1^	1.00	1.01 (0.75–1.49)	1.14 (0.83–1.57)	0.82 (0.54–1.24)	0.31	0.75
CVD (*n*, %)	132 (9.9)	323 (24.1)	527 (39.4)	356 (26.6)		
Deaths; person-years	26; 2149.9	33; 5563.6	41; 9134.4	15; 6294.5		
Multivariable ^1^	1.00	1.01 (0.69–1.47)	1.16 (0.82–1.66)	0.75 (0.47–1.20)	0.25	0.47
Cancer (*n*, %)	116 (8.9)	314 (24.1)	518 (39.7)	357 (27.4)		
Deaths; person-years	10; 2003.3	24; 5457.3	32; 9044.9	16; 6268.5		
Multivariable ^1^	1.00	1.86 (0.85–4.10)	1.89 (0.87–4.08)	1.83 (0.77–4.31)	0.35	0.23

Abbreviations: CI: confidence interval; CVD: cardiovascular disease. ^1^ HR (95% CI) for Cox regression model adjusted for age (continuous), sex, educational level (<Primary, ≥Primary), BMI (<25, 25.0–29.9, ≥30), sleeping time (hours/day), smoking habit (current, past, and never), self-reported diabetes (no/yes), hypertension (no/yes), and TV-watching (hours/day). ^2^ *p*-value from likelihood ratio test.

## Data Availability

The data presented in this study are available on request from the corresponding author. The data are not publicly available due to confidentiality and ethical reasons.
